# STV-FSANet: Track-Level Spatio-Temporal Verification for Fire and Smoke Alarm Validation in Video Surveillance

**DOI:** 10.3390/s26154970

**Published:** 2026-08-05

**Authors:** Deepak Ghimire, Donghoon Kim, Yeonho Jo, Eunhee Lee, Sunghwan Jeong, Byoungjun Kim

**Affiliations:** AI Application Research Center, Korea Electronics Technology Institute, Jeonju 54853, Republic of Korea; deepak@keti.re.kr (D.G.); clickmiss123@keti.re.kr (D.K.); geijin0821@keti.re.kr (Y.J.); ilmare7@keti.re.kr (E.L.); shjeong@keti.re.kr (S.J.)

**Keywords:** fire detection, smoke detection, video surveillance, temporal verification, false alarm suppression, recurrent neural network, GRU, track-based analysis

## Abstract

Generating early and reliable fire/smoke alarms from real-world video surveillance remains challenging because visually ambiguous patterns such as sunlight, reflections, clouds, mist, steam, and illumination changes often trigger unstable frame-level false alarms. This paper presents the Spatio-Temporal Verification Network for Fire and Smoke Alarm Validation (STV-FSANet), a detect–track–verify framework that first localizes candidate fire/smoke regions, associates them into temporal tracks, and then verifies the observed sequence of each active track online as fire, smoke, or false fire/smoke. The verifier combines a primary appearance stream from cropped candidate regions with lightweight geometric cues derived from bounding-box position, scale, motion, and short-term fluctuation. A dual-branch GRU models long-term track history, while recent temporal pooling emphasizes newly observed evidence for streaming decisions. To support temporal learning, we construct the Fire–Smoke Alarm Verification (FSAV) Tracklet Dataset from 1347 source videos, yielding 58,733 parent tracks and 2.06 million annotated track frames. The best matched-context STV-FSANet achieves 97.09% test accuracy and 95.81% macro-F1, and both shorter-context models are within 0.5 percentage points of their final prefix metrics by 1.0 s. The results indicate that compact temporal evidence is more useful than simply accumulating longer 96-frame histories. The proposed model also rejects false fire/smoke tracklets with 90.2% recall, demonstrating the value of explicit hard-negative modeling, while the decoupled design allows the verifier to be reused with future detector backbones. TensorRT FP16 deployment reaches 109.55 frames/s on an RTX 4060 Laptop GPU and 39.51 frames/s on a Jetson AGX Orin DevKit.

## 1. Introduction

Automatic fire and smoke detection using surveillance cameras has attracted increasing attention because of its importance for public safety, industrial monitoring, forest fires, transportation safety, and smart city applications [1,2,3,4,5,6]. Compared with point sensors, camera-based systems provide wider spatial coverage and earlier visual warning, especially outdoors. However, real surveillance remains difficult because fire and smoke vary strongly with distance, weather, illumination, viewpoint, and scene context. Non-fire phenomena such as sunlight, reflections, haze, steam, clouds, and artificial lighting can also create fire/smoke-like patterns and trigger false alarms.

Recent advances in deep learning have improved fire and smoke recognition at the video level, ranging from CNN-based video fire analysis [7,8,9] to detector-based smoke and flame localization [10,11,12,13,14,15]. Recent studies also emphasize lightweight real-time and scenario-adaptive systems for operational surveillance video [16,17,18,19]. Nevertheless, many approaches still rely on decisions from single images or independent frames. This is problematic for alarm generation: a fire-like or smoke-like region in one frame may be transient or inconsistent, while real events usually show sustained presence, motion continuity, gradual spatial growth, or characteristic instability. A reliable system should therefore locate candidate regions and verify whether their temporal behavior is consistent with real fire or smoke.

Motivated by this observation, we propose *STV-FSANet*, a spatio-temporal verification framework that separates full-frame candidate localization from track-level alarm validation. A detector localizes boxes, temporal association forms candidate sequences, and STV-FSANet verifies each sequence as fire, smoke, or false fire/smoke from its appearance and trajectory evidence. A unified spatio-temporal detector is also a valid design; the staged formulation reflects this study’s focus on candidate-level false-alarm suppression and allows the verifier to be updated independently. Detector class labels are unused, but a sufficiently localized and associated candidate box is required.

To achieve this goal, we model temporal evidence through a primary appearance branch and a lightweight geometry branch. The appearance branch captures *visual temporal evolution* from cropped candidate regions, while the geometry branch contributes low-cost cues from tracked box position, size, motion, and short-term fluctuation. This design follows recent temporally aware video fire/smoke models showing the importance of sequential evidence beyond isolated frame-level classification [20,21,22,23,24]. The two streams are processed by a dual-branch GRU-based verifier, where recurrent states model track history and recent temporal pooling emphasizes newly accumulated evidence. Thus, verification can start once a candidate track becomes stable and can be progressively refined as the track grows.

Another challenge is the lack of a benchmark suited to temporal fire/smoke verification. Public datasets differ in format, scale, annotation style, and target scenario, and many provide only frame-level labels or partial event categories [25,26,27]. We therefore construct the *Fire–Smoke Alarm Verification (FSAV) Tracklet Dataset*, a unified benchmark from five public source collections and one collection obtained directly from its original authors. Detector-guided candidate tracks are manually reviewed and labeled as fire, smoke, or false fire/smoke, enabling track-level temporal learning and evaluation under diverse real-world scenarios.

**Figure 1:**
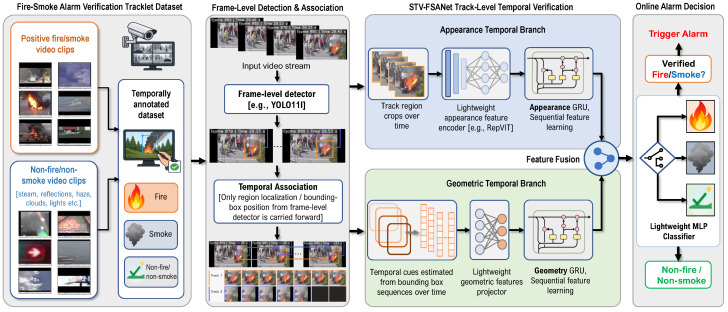
Overall STV-FSANet workflow for track-level fire and smoke alarm validation. Source videos are curated into the FSAV Tracklet Dataset using detector-guided candidate tracks. During inference, frame-level candidate regions are associated into temporal tracks, whose observed sequences are verified online as fire, smoke, or false fire/smoke using accumulated appearance and geometric evidence. This keeps candidate generation separate from reusable temporal validation.

Figure 1 summarizes the proposed workflow. Positive fire/smoke clips and hard-negative videos are used to build the FSAV Tracklet Dataset. At inference time, a frame-level detector generates candidate regions, temporal association converts them into tracks, and the dual-branch verifier produces the final fire, smoke, or false fire/smoke alarm decision. The same decoupled design keeps fast localization separate from reusable temporal validation.

The main contributions are summarized as follows:

- We propose STV-FSANet, a spatio-temporal detect–track–verify framework for fire and smoke alarm validation in real-world video surveillance. It combines lightweight candidate-region generation with modular track-level temporal verification to improve alarm reliability.
- We design a dual-branch GRU-based temporal verifier that uses visual appearance evolution as the primary temporal signal and augments it with lightweight geometric motion and scale cues to classify tracked candidates as fire, smoke, or false fire/smoke.
- We introduce a streaming-oriented recurrent verification strategy that combines long-term hidden-state memory with recent temporal evidence, enabling early prediction and progressive online decision refinement for long and variable-length tracks.
- We construct the FSAV Tracklet Dataset with 1347 source videos, resulting in 58,733 parent tracks and 2.06 million annotated track frames from five public sources and the author-provided Kim–Lee source.
- We demonstrate that the proposed framework achieves up to 97.09% test accuracy and 95.81% macro-F1, reaches near-final prefix metrics by 1.0 s, and improves false-alarm suppression while preserving the efficiency required for practical edge deployment.

The remainder of this paper is organized as follows. Section 2 reviews related fire/smoke detection methods, temporal verification strategies, and public video datasets. Section 3 presents the proposed framework, Section 4 describes the FSAV Tracklet Dataset, Section 5 reports experiments and runtime analysis, Section 6 discusses practical strengths and limitations, and Section 7 summarizes the main findings and deployment implications.

## 2. Related Work

Vision-based fire and smoke detection has moved from handcrafted visual features to deep spatio-temporal models. Reliable real-time alarms remain challenging, however, because visually similar non-fire patterns can produce unstable frame-level decisions. The work most related to this study falls into three areas: frame-level fire/smoke detection, temporal modeling for reliability, and public video datasets for benchmarking. STV-FSANet builds on these areas while focusing on verified alarms from tracked candidate regions rather than direct per-frame decisions.

### 2.1. Fire and Smoke Detection in Images and Videos

Early vision-based systems used manually designed color, texture, motion, contour, and spatio-temporal cues to detect flames or smoke. Representative examples include machine-vision flame/smoke detection [1]; integrated surveillance fire detection [28]; wildfire smoke detection with spatio-temporal features and random forests [29]; expert fusion of color, shape, and motion cues [30]; smoke detection using image energy and color [31]; texture-based flame detection for robotic platforms [32]; and multi-stage or LBP-sequence smoke alarm methods [33,34]. These methods highlighted dynamic visual cues but were sensitive to illumination changes, clutter, haze, clouds, and camera-specific appearance shifts.

Deep learning has improved fire/smoke recognition by replacing handcrafted features with learned spatial representations. CNN-based video fire detection by Kim and Lee [7] and their Bayesian fusion framework [8] showed the value of learned visual features, while later methods expanded frame-level localization through integrated CNN and detector-based pipelines. Examples include UFS-Net [9], EfficientDet-based long-range wildfire smoke detection [10], EdgeFireSmoke and EdgeFireSmoke++ for lightweight real-time recognition [11,12], additive neural models for forest-fire recognition [35], vision-transformer-based smoke detection [36], YOLO-based fire/smoke localization [13], and benchmark-oriented smoke detection on camera networks [25,26]. Recent surveys [2,3,4,5,6] show that image classification, object localization, and segmentation remain the dominant formulations.

Despite these advances, detector outputs are often used directly for alarm decisions. Many false positives are visually plausible in single frames but temporally unstable, so improved spatial detection alone does not fully solve reliable alarm generation. This motivates the temporal verification strategy adopted in this paper.

### 2.2. Temporal Verification and False-Alarm Suppression

False-alarm suppression has motivated a second line of work that explicitly uses temporal information. Traditional and transitional methods have used motion flicker, blurred edges, temporal texture changes, local extremal region tracking, domain adaptation, and adaptive decision fusion to distinguish persistent smoke from transient artifacts [21,29,30,37,38,39]. Deep models extend this idea through sequential and spatio-temporal learning. Xie et al. combined motion-flicker dynamics with deep static features [20]; Huo et al. introduced 3DVSD for end-to-end 3D convolutional smoke localization [22]; FireMatch studied semi-supervised video fire detection under limited labels [40]; and STENet [23], TIRNet [24], and FOCUS [17] model spatial structure and temporal evolution for complex scenes.

These studies show that temporal evidence is important, but many methods still operate as clip-level recognizers or end-to-end video detectors in which temporal reasoning is tied to the detection backbone. Our objective is different: STV-FSANet validates temporally associated candidate regions with a separate verifier. Lightweight localization can maintain speed and sensitivity, while the verifier focuses on rejecting fire/smoke-like false positives using candidate crops and track geometry rather than detector logits. The remaining gap is how to organize temporal evidence for online alarm validation over variable-length tracks. STV-FSANet addresses this space by combining appearance evolution, lightweight geometric cues, recurrent memory, and recent evidence accumulation in a detect–track–verify framework.

### 2.3. Source Fire/Smoke Video Datasets

Public datasets have supported progress in the field, but they remain heterogeneous in supervision style and temporal structure. Classical benchmarks such as *FIRESENSE* [41], *KMU Fire & Smoke* [29], *Bilkent VisiFire* [42], and *Furg Fire Dataset* [32] remain useful for robustness testing, smoke/flame diversity, and hard-negative analysis. More recent resources have expanded scale and annotation quality, especially for wildfire smoke, including *SmokeNet-ProjectX* [25], the Kim–Lee video fire datasets used in [7,8], the *Nemo* benchmark [26], the large-scale *PYRONEAR-2025* benchmark [27], and the multimodal indoor-fire dataset *MmodalFire* [43]. Recent reviews also emphasize that dataset quality, annotation consistency, and realistic hard negatives remain major bottlenecks to progress in the field [3,4,5,6].

For temporal alarm validation, however, the available datasets are fragmented. Some emphasize only flame or smoke, some provide only frame-wise labels, and others use dataset-specific sequence formats. They generally do not provide a unified false fire/smoke category or a consistent track-level formulation for validating candidate tracks after detection and association. Therefore, the public datasets used here are curated as source components of the FSAV Tracklet Dataset, described in Section 4, which is designed for three-class track-level alarm validation.

## 3. Methods

### 3.1. Overview of the Proposed Framework

As illustrated in Figure 1, a detector localizes candidate boxes, temporal association forms tracks, and STV-FSANet updates each active track’s fire, smoke, or false fire/smoke decision. The detector and tracker are fixed upstream utilities; their comparative design, training, and ablation are outside this verifier-focused study, although their settings and end-to-end pipeline efficiency are reported for reproducibility. STV-FSANet uses associated crops and box trajectories, not detector labels, but missed, poorly localized, or fragmented candidates can still reduce end-to-end coverage.

To model temporal evidence, the verification stage employs two branches. The primary branch captures visual temporal evolution by extracting appearance features from cropped candidate regions across time. The auxiliary branch captures lightweight geometric cues from tracked bounding boxes, including normalized position, scale, area, motion, and short-term fluctuation patterns. These two temporal streams are then processed by a dual-branch GRU-based verifier, where recurrent hidden states encode long-term track history and recent temporal pooling emphasizes newly observed evidence. This combination preserves temporal context while adapting to the recent track state.

This design is driven by surveillance practice: a candidate may appear ambiguous at first and become discriminative only after several frames. Verification begins once a track becomes sufficiently stable, and the fire, smoke, or false fire/smoke decision is updated online. This improves alarm reliability by suppressing transient false positives while retaining early response.

To support this framework, we constructed the FSAV Tracklet Dataset by curating five public video sources and the Kim–Lee collection obtained directly from its original authors into fire, smoke, and false fire/smoke track categories. This benchmark allows the verifier to be trained and evaluated under diverse conditions while keeping the detector replaceable and the verifier reusable.

**Terminology.** Throughout this paper, *detection* means frame-level localization of a candidate bounding box. A *track* is the full temporally associated trajectory of one candidate in a source video; an incomplete trajectory maintained during streaming is called an active track. A *tracklet* (or subtrack) is a bounded segment derived from a parent track and used as one dataset instance. A *sequence* is the ordered set of sampled crop and geometry observations supplied to the verifier from a tracklet or active track. *Classification* denotes the model operation that maps this sequence to fire, smoke, or false fire/smoke, whereas *verification* denotes its operational use to accept a fire/smoke alarm candidate or reject a false one. *Temporal context* is the elapsed duration or maximum number of sampled observations made available to the verifier, and *temporal evidence* is the appearance and bounding-box-derived geometry actually accumulated within that context. A *prefix* is an initial portion of a sequence used for early online prediction.

### 3.2. Frame-Level Detection and Temporal Association

The first stage performs frame-level candidate localization. A lightweight detector is trained using an image-based fire/smoke dataset to propose fire- or smoke-like regions within each frame. In both experimental and deployment benchmarks, we used a separately trained application-specific YOLO11l model from Ultralytics YOLO11 [44] because it provided a practical balance between candidate localization and throughput. The goal is efficient candidate detection with high recall, even if some false positives are generated. In the proposed system, the detector does not determine the final alarm state; it only localizes candidate regions that are later analyzed by STV-FSANet for alarm validation.

For an input video, the detector is applied independently to each frame to obtain a set of candidate bounding boxes. Let the set of detections at frame *t* be

(1) $$D^{\left(t\right)}=\left\{d_{1}^{\left(t\right)},d_{2}^{\left(t\right)},\ldots ,d_{N_{t}}^{\left(t\right)}\right\},$$

where $N_{t}$ is the number of candidate detections at frame *t*, and each detection is represented by a bounding box

(2) $$d_{i}^{\left(t\right)}=\left(x_{1}^{\left(t\right)},y_{1}^{\left(t\right)},x_{2}^{\left(t\right)},y_{2}^{\left(t\right)}\right).$$

These frame-level detections may belong to true fire, true smoke, or fire/smoke-like false positives. Because multiple events may appear simultaneously in the same frame, all detections are treated as independent candidates during temporal association.

Temporal association transforms frame-level detections into candidate tracks. Let

(3) $$T=\left\{\tau_{1},\tau_{2},\ldots ,\tau_{M}\right\}$$

denote the set of candidate tracks formed by associating the detector outputs, where each track $\tau_{m}$ is defined as an ordered sequence of detections belonging to the same evolving region:

(4) $$\tau_{m}=\left\{d_{m}^{\left(t_{1}\right)},d_{m}^{\left(t_{2}\right)},\ldots ,d_{m}^{\left(t_{L}\right)}\right\},$$

with *L* denoting the track length. Let $b_{m}^{\left(t-1\right)}$ be the latest box of active track *m* and $d_{i}^{\left(t\right)}$ a current detection. Their association score is

(5) $$s_{mi}^{\left(t\right)}=\left\{\begin{array}{cc} \frac{|b_{m}^{\left(t-1\right)}\cap d_{i}^{\left(t\right)}|}{|b_{m}^{\left(t-1\right)}\cup d_{i}^{\left(t\right)}|}, & \mathrm{class}\text{-}\mathrm{agnostic},\;\mathrm{or}\;c_{m}=c_{i},\\ -\infty , & \mathrm{otherwise}\;\mathrm{in}\;\mathrm{class}\text{-}\mathrm{aware}\;\mathrm{mode}, \end{array}\right.$$

where $c_{m}$ and $c_{i}$ are detector class IDs. Eligible pairs are sorted by decreasing score and accepted greedily under one-to-one assignment:

(6) $$M^{\left(t\right)}={\mathrm{Greedy}}_{1:1}\left(\{\left(m,i\right):s_{mi}^{\left(t\right)}\geq \tau_{\mathrm{IoU}}\}\right).$$

A match updates the track box; every unmatched detection receives a new monotonically increasing track ID. For missed-frame count $q_{m}^{\left(t\right)}$,

(7) $$q_{m}^{\left(t\right)}=\left\{\begin{array}{cc} 0, & m\;\mathrm{is}\;\mathrm{matched},\\ q_{m}^{\left(t-1\right)}+1, & m\;\mathrm{is}\;\mathrm{unmatched}, \end{array}\right.\;\mathrm{retire}\;\mathrm{track}\;m\;\mathrm{if}\;q_{m}^{\left(t\right)}>M_{\mathrm{miss}}.$$

Here, $\tau_{\mathrm{IoU}}$ is the association threshold, and $M_{\mathrm{miss}}$ is the maximum number of consecutive missed frames for which a track is retained. Confidence, appearance embeddings, motion prediction, and re-identification are not used for matching, and detector labels are not supplied to STV-FSANet. The tracker is not intended to solve generic multi-object tracking in complex semantic scenes; its role is to preserve candidate-region continuity for subsequent verification.

Temporal tracks help distinguish fire and smoke from visually similar transient highlights or reflections that lack coherent appearance, motion, and spatial evolution. The verifier therefore processes variable-length candidate tracks rather than isolated detections. Each track comprises synchronized crop and bounding-box sequences for appearance and geometric modeling, respectively. Because detector logits and labels are not used, the verifier remains largely detector-agnostic, provided that candidates are localized and associated over time.

### 3.3. Lightweight Geometric Temporal Cues

For each tracked candidate region at frame *t*, let the corresponding bounding box be $b^{\left(t\right)}=\left(x_{1}^{\left(t\right)},y_{1}^{\left(t\right)},x_{2}^{\left(t\right)},y_{2}^{\left(t\right)}\right)$ in an image of width *W* and height *H*. The geometric branch analyzes bounding-box properties as a low-cost auxiliary stream describing where the candidate appears, how its scale and shape evolve, and whether its motion is stable or fluctuating. All geometric features are normalized by the image dimensions so that the same descriptor can be used across videos with different resolutions. Table 1 summarizes the cues used to construct the geometric input at each frame and organizes them into three fixed, non-overlapping feature families.

For the first frame in a tracklet, where no previous observation is available, the temporal difference terms and motion cue are initialized to zero. Short-term fluctuation terms are computed from the available history within the current tracklet. The resulting 18-dimensional geometric feature vector is

(8) $$\begin{array}{cc} g^{\left(t\right)}= & [cx_{n}^{\left(t\right)},cy_{n}^{\left(t\right)},w_{n}^{\left(t\right)},h_{n}^{\left(t\right)},area_{n}^{\left(t\right)},aspect^{\left(t\right)},dcx^{\left(t\right)},dcy^{\left(t\right)},dw^{\left(t\right)},dh^{\left(t\right)},darea^{\left(t\right)},daspect^{\left(t\right)},\\ \; & motion\_mag^{\left(t\right)},upward\_motion^{\left(t\right)},std\_area_{k}^{\left(t\right)},std\_cx_{k}^{\left(t\right)},std\_cy_{k}^{\left(t\right)},std\_aspect_{k}^{\left(t\right)}]. \end{array}$$

This descriptor summarizes spatial configuration, temporal variation, motion trend, and recent fluctuations, and is used as the auxiliary geometric input sequence to the proposed temporal verification module. Because these cues are computed from detector boxes, accurate and temporally stable localization yields more faithful geometric trajectories, whereas localization jitter or track fragmentation can degrade this auxiliary signal.

### 3.4. Dual-Branch GRU Verifier

**Figure 2:**
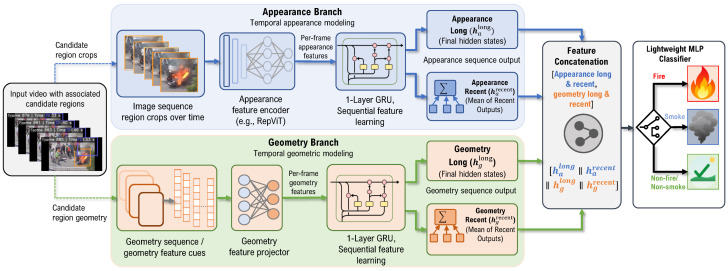
Overview of the proposed dual-branch GRU verifier. In the appearance branch, input crops from the tracked regions are processed by the RepViT [45] encoder and passed through a GRU to generate both the last hidden state and an average of recent outputs. In parallel, the geometric branch passes bounding-box features through a dedicated projector and GRU, followed by the same pooling. All summary vectors are concatenated and passed through a multilayer perceptron to produce a final class prediction.

Given a candidate track produced by temporal association across video frames, the proposed *Dual-Branch GRU Verifier* performs track-level temporal verification to classify the tracked candidate region as *fire*, *smoke*, or *false fire/smoke*. The verifier is designed to exploit two coordinated forms of temporal evidence. The primary branch models the visual evolution of cropped candidate regions, while the auxiliary geometry branch contributes low-dimensional geometric cues derived from the tracked bounding boxes over time. These two streams are processed in parallel and integrated to produce the final track-level prediction, as shown in Figure 2.

#### 3.4.1. Input Representation

For a tracklet of length *T*, the proposed verifier receives two synchronized temporal inputs. The first is the cropped image sequence

(9) $$X=\{x^{\left(1\right)},x^{\left(2\right)},\ldots ,x^{\left(T\right)}\},$$

where $x^{\left(t\right)}\in R^{3\times H\times W}$ denotes the cropped candidate region at frame *t*. The second is the geometric feature sequence

(10) $$G=\{g^{\left(1\right)},g^{\left(2\right)},\ldots ,g^{\left(T\right)}\},$$

where $g^{\left(t\right)}\in R^{18}$ is the geometric temporal feature descriptor defined in (8).

Because track lengths vary across samples, the verifier is designed to support variable-length temporal sequences. During training, all sequences in a mini-batch are padded to the maximum batch length, and before recurrent processing, the corresponding valid lengths are used to pack the temporal inputs. This allows the GRU branches to operate only on valid observations and avoids extra computation over padded time steps.

#### 3.4.2. Appearance Branch

The appearance branch captures the temporal evolution of the visual content inside the tracked candidate region.

**Per-frame appearance encoding.** Each cropped image $x^{\left(t\right)}$ is resized to a standard size and then passed through a lightweight visual encoder, RepViT [45], denoted by $f_{\mathrm{enc}}\left(\cdot \right)$, to produce a compact appearance feature
  (11) $$a^{\left(t\right)}=f_{\mathrm{enc}}\;\left(x^{\left(t\right)}\right),\;a^{\left(t\right)}\in R^{d_{a}},$$
  where $d_{a}$ is the appearance feature dimension. RepViT [45] is used for its compact mobile-oriented accuracy–efficiency trade-off; however, other backbones such as MobileNetV2 [46] or another efficient image encoder could be used without changing the temporal verification module. The appearance sequence for the full input track to the verifier is therefore written as
  (12) $$A=\{a^{\left(1\right)},a^{\left(2\right)},\ldots ,a^{\left(T\right)}\}.$$
**Temporal appearance modeling.** To capture long-term visual evolution, the encoded sequence is processed by a GRU:
  (13) $$h_{a}^{\left(t\right)}={\mathrm{GRU}}_{a}\;\left(a^{\left(t\right)},h_{a}^{\left(t-1\right)}\right),$$
  where $h_{a}^{\left(t\right)}\in R^{h_{a}}$ is the hidden state at frame *t*. The final hidden state
  (14) $$h_{a}^{\mathrm{long}}=h_{a}^{\left(T\right)}$$
  serves as a long-term appearance memory summarizing the history of the candidate track.
**Recent appearance pooling.** In addition to long-term memory, the appearance branch computes a short-term summary from the most recent *K* valid time steps:
  (15) $$h_{a}^{\mathrm{recent}}=\frac{1}{K^{\prime }}\sum_{t=T-K^{\prime }+1}^{T}h_{a}^{\left(t\right)},$$
  where $K^{\prime }=\min\left(K,T\right)$. The parameter *K* sets the maximum number of recent temporal steps considered. This pooled representation emphasizes newly accumulated evidence that is often more informative for the current track state and is suitable for online decision-making.

#### 3.4.3. Geometric Branch

The geometric branch models the geometric temporal evolution from the tracked bounding boxes.

**Per-frame geometric projection.** At each temporal step, the raw geometric descriptor $g^{\left(t\right)}\in R^{18}$ is first projected into a learned embedding space using a lightweight multilayer perceptron:
  (16) $$z^{\left(t\right)}=f_{\mathrm{geo}}\;\left(g^{\left(t\right)}\right),\;z^{\left(t\right)}\in R^{d_{g}},$$
  where $d_{g}$ is the geometric embedding dimension. The projected geometric sequence is then written as
  (17) $$Z=\{z^{\left(1\right)},z^{\left(2\right)},\ldots ,z^{\left(T\right)}\}.$$
**Temporal geometric modeling.** The projected geometric sequence is processed by a second GRU:
  (18) $$h_{g}^{\left(t\right)}={\mathrm{GRU}}_{g}\;\left(z^{\left(t\right)},h_{g}^{\left(t-1\right)}\right),$$
  where $h_{g}^{\left(t\right)}\in R^{h_{g}}$ is the hidden state at frame *t*. The final hidden state
  (19) $$h_{g}^{\mathrm{long}}=h_{g}^{\left(T\right)}$$
  acts as a long-term geometric summary of the spatial and motion evolution of the track over time.
**Recent geometric pooling.** As in the appearance branch, a recent pooled summary is computed over the last *K* valid frames to preserve sensitivity to newly observed motion and shape changes:
  (20) $$h_{g}^{\mathrm{recent}}=\frac{1}{K^{\prime }}\sum_{t=T-K^{\prime }+1}^{T}h_{g}^{\left(t\right)},$$
  where $K^{\prime }=\min\left(K,T\right)$. This branch is intentionally kept lightweight and is used to provide complementary temporal evidence rather than to replace the appearance modeling branch.

#### 3.4.4. Feature Fusion and Classification

The final representation of an input track is obtained by concatenating long-term and recent temporal features from both branches:

(21) $$f=\left[h_{a}^{\mathrm{long}}\;∥\;h_{g}^{\mathrm{long}}\;∥\;h_{a}^{\mathrm{recent}}\;∥\;h_{g}^{\mathrm{recent}}\right],$$

where ‖ denotes feature concatenation.

This fused feature descriptor is then passed through a lightweight multilayer classifier:

(22) $$\hat{y}=f_{\mathrm{cls}}\left(f\right),$$

where $\hat{y}\in R^{3}$ contains the logits for the three target classes

{fire,smoke,falsefire/smoke}.

The final prediction is obtained as

(23) $$\hat{c}=\arg\max_{c}{\hat{y}}_{c}.$$

During training, the model is optimized using the standard cross-entropy loss:

(24) $$L_{\mathrm{cls}}=-\sum_{c=1}^{3}y_{c}\log\left(\mathrm{softmax}{\left(\hat{y}\right)}_{c}\right),$$

where $y_{c}$ is the ground-truth one-hot label and $\hat{y}$ denotes the predicted logit vector.

#### 3.4.5. Long-Term Memory and Short-Term Adaptation

A core design principle of the verifier is the integration of long-term recurrent memory with short-term adaptation. The hidden states $h_{a}^{\mathrm{long}}$ and $h_{g}^{\mathrm{long}}$ provide concise summaries of the complete temporal history of the candidate track. However, relying only on the final recurrent state may make the model respond too slowly to changes in current track behavior. Recent temporal pooling is therefore incorporated in both branches so the verifier remains sensitive to newly accumulated evidence while preserving broader context. This balances stability and responsiveness in streaming surveillance, where early decisions must be updated as new observations arrive.

#### 3.4.6. Online Inference

Although the verifier is trained using tracklet sequences, the same model is directly applicable to streaming inference. As new detections are associated with an active track, both the appearance and geometric branches incrementally update their GRU hidden states. At the same time, recent temporal pooling summarizes only the latest *K* valid observations, enabling the verifier to refine its decision online without the need to store the complete track history explicitly. This design is efficient and well-suited for real-time surveillance, where predictions must begin early, adapt as new evidence accumulates, and remain robust against transient false positives.

### 3.5. Online Verification and Alarm Decision

During deployment, the detector provides candidate regions in each frame and temporal association maintains active tracks. STV-FSANet is applied to the currently observed sequence prefix of each active track, so the output is an online estimate of the current alarm state rather than a label assigned only after the track has ended. This allows the verifier to start early and revise its decision as additional evidence becomes available. When multiple tracks are active in the same frame, each is verified independently; for efficient implementation, the eligible sequence prefixes are stacked and processed as a batch during inference.

Let $\tau_{1:t}=\left\{\left(x^{\left(1\right)},g^{\left(1\right)}\right),\ldots ,\left(x^{\left(t\right)},g^{\left(t\right)}\right)\right\}$ denote the observed sequence prefix of an active track, where $x^{\left(t\right)}$ is the cropped candidate region and $g^{\left(t\right)}$ is the corresponding geometric descriptor. Verification is delayed until a minimum number of observations is available. With $T_{\min}$ denoting this minimum verification length, no track-level alarm decision is produced when $t<T_{\min}$. Once the prefix satisfies

(25) $$t\geq T_{\min},$$

the verifier outputs a logit vector

(26) $${\hat{y}}^{\left(t\right)}=\left[{\hat{y}}_{\mathrm{fire}}^{\left(t\right)},\;{\hat{y}}_{\mathrm{smoke}}^{\left(t\right)},\;{\hat{y}}_{\mathrm{false}}^{\left(t\right)}\right],$$

which is converted into class probabilities through the softmax function:

(27) $$p^{\left(t\right)}=\mathrm{softmax}\;\left({\hat{y}}^{\left(t\right)}\right).$$

The current predicted class is then given by

(28) $${\hat{c}}^{\left(t\right)}=\arg\max_{c}p_{c}^{\left(t\right)},$$

where c∈{fire,smoke,false} and the false class denotes false fire/smoke. The predicted state is updated whenever the active track receives a new observation. Tracks classified as fire or smoke are treated as verified alarm candidates, whereas tracks classified as false fire/smoke are rejected. Short-lived tracks that disappear before satisfying $T_{\min}$ are discarded without an alarm decision. Thus, the alarm decision is based on accumulated temporal evidence from the active track rather than on an isolated frame-level detection.

## 4. Fire–Smoke Alarm Verification (FSAV) Tracklet Dataset

### 4.1. Dataset Sources and Annotation Strategy

Temporal fire/smoke verification lacks a standardized benchmark that jointly supports *fire*, *smoke*, and *false fire/smoke* track-level learning under realistic surveillance conditions. Existing public datasets differ in scene type, annotation format, semantic scope, and temporal structure. We address this by curating five public collections and one author-provided collection into the FSAV Tracklet Dataset, a unified track-oriented benchmark. The source videos are summarized in Table 2, and the final statistics are reported in Table 3.

The only source obtained directly from its original authors was the Kim–Lee collection; no source videos were privately recorded by the present authors. It contributes 617 of 1347 selected videos (45.81%), 26,623 of 58,733 parent tracks (45.33%), and 987,508 of 2,064,171 annotated track frames (47.84%). The 617 selected videos comprise 107 fire clips, 247 benign smoke/steam clips, and 263 non-fire clips. The source documentation further identifies flame, smoke, and fire scenes, together with hard non-fire conditions such as chimney smoke, sunsets, clouds, and other visually irrelevant objects [7,8].

The parent tracks and their derived tracklets are organized into three target categories: *fire*, *smoke*, and *false fire/smoke*. The last category contains detector-proposed regions that survived temporal association and were manually verified as neither fire nor smoke; arbitrary background crops were not added. It includes clouds, fog/haze, steam-like regions, reflections, glare, bright light sources, and other detector failure modes and is essential for verification-oriented false-positive suppression.

For Bilkent VisiFire, 29 fire/smoke clips (13 fire and 16 smoke) were selected. Detector proposals from one smoke clip also yielded two hard-negative regions labeled as false fire/smoke during manual verification.

The original datasets are not directly usable because their annotations generally do not match the three-class track-level formulation. When source annotations are available, we combine them with candidate boxes from a separately trained frame-level detector. When usable annotations are absent, tracks are generated from detector outputs alone. All tracks are then manually reviewed with a custom tracklet-review tool and organized into temporally ordered samples with bounding boxes and final track-level labels.

**Figure 3:**
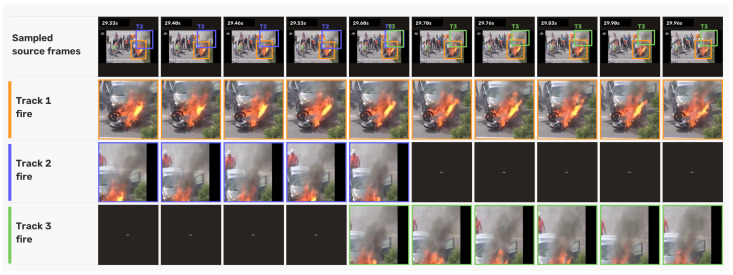
Single-video example of temporal tracklet construction. The first row shows sampled source frames with color-coded final track boxes, and each following row shows the corresponding 128×128 crops for one track identity. Track identities are renumbered locally for visualization. Blank cells indicate sampled frames in which that track is not present. Timestamp overlays are shown to two decimal places so that adjacent sampled source frames are uniquely identified.

**Figure 4:**
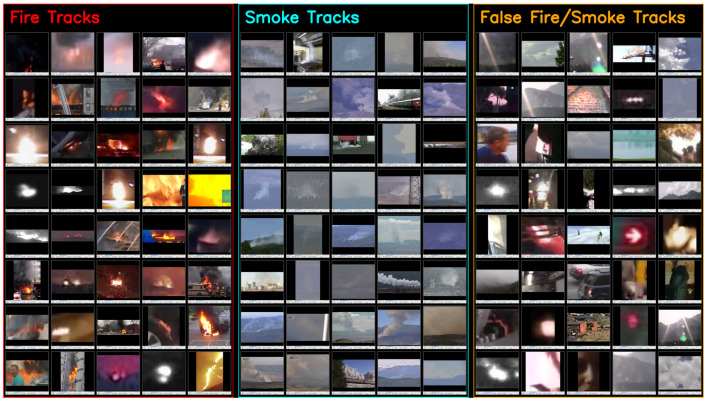
Qualitative montage of FSAV track crops. The left, middle, and right panels show *fire*, *smoke*, and *false fire/smoke* tracks, respectively. The false panel includes diverse detector-generated confounders. The montage illustrates visual variation rather than class or subtype frequencies.

Figure 3 shows how final tracklets are obtained from a single source video. The top row samples source frames and overlays final tracking boxes with consistent colors. Each subsequent row shows one track identity across the same sampled time points and the standardized 128×128 crops used as verifier inputs. Figure 4 then provides a dataset-level montage of representative crops from the three target categories, with the false panel showing fire/smoke-like hard negatives from diverse detector-triggered scenes.

### 4.2. Track Construction and Label Definition

The temporal fire/smoke verifier operates on candidate tracks rather than isolated frame-level detections, so all dataset annotations are expressed as track-level temporal samples. A track is defined as

(29) $$\tau_{m}=\left\{b_{m}^{\left(t_{1}\right)},b_{m}^{\left(t_{2}\right)},\ldots ,b_{m}^{\left(t_{L}\right)}\right\},$$

where $b_{m}^{\left(t\right)}$ denotes the bounding box of track *m* at frame *t*, and *L* is the track length.

Each track is assigned one of three labels:

(30) $$y_{m}\in \left\{\mathrm{fire},\;\mathrm{smoke},\;\mathrm{false}\;\mathrm{fire}/\mathrm{smoke}\right\}.$$

A *fire* track represents a temporally consistent fire/flame region, a *smoke* track represents a temporally consistent smoke region, and a *false fire/smoke* track represents a visually confusing region that does not correspond to a true fire or smoke event. This third class explicitly models practical false positives that must be rejected during deployment. Any candidate region containing visible flame is labeled as fire even when smoke co-occurs, whereas the smoke label is reserved for regions without visible flame. This mutually exclusive alarm-priority rule avoids delaying a flame alarm; it does not encode the relative fire/smoke area. Because mixture status was not stored as a separate annotation attribute, a complete mixed-track proportion cannot be recovered reliably from the current single-label inventory.

Track construction is performed by combining available source annotations with detector-based proposals. When suitable source annotations are available, they guide localization and labeling. When annotations are absent or insufficient for our three-class setting, candidate regions are generated from the frame-level fire/smoke detector. These frame-wise detections are then associated across time to form candidate tracks, and all resulting tracks are manually verified using our custom tracklet-review tool. The final annotation format stores, for each video, a set of track identities, frame indices, bounding boxes, and verified track-level labels.

For each context duration d∈{3.2,6.4,9.6} s, the corresponding dataset build limits a subtrack to $L_{d}=\mathrm{round}\left(fd\right)$ native track observations, where *f* is the source-video frame rate. A parent track with more than $L_{d}$ observations yields up to three unique, potentially overlapping windows anchored at its beginning, midpoint, and end; no separate short remainder is created. Every window inherits the manually verified parent-track label without relabeling. At data loading, the target rate of 10 frames per second (fps) and tail truncation limit the model input to 32, 64, or 96 observations, respectively, and sequences with fewer than five sampled observations are excluded. During training, the verifier can also consume variable-length prefixes of a tracklet, which enables early-decision learning and supports progressive decision refinement during online inference.

### 4.3. Dataset Split and Statistics

Table 3 reports the 58,733 parent tracks before duration-specific segmentation; it therefore does not correspond to any single context length. For each context, the derived tracklets are stratified into a nominal 65%/15%/20% train/validation/test partition. Table 4 distinguishes the resulting nominal subtracks from the sequences remaining after 10 fps sampling and the five-observation eligibility rule.

To prevent identical verifier observations and related windows across evaluation boundaries, a test tracklet is excluded if any sampled candidate-region observation, identified by its source dataset, video, parent track, frame index, and bounding box, also occurs in training or validation, or if another window from the same parent track occurs in either split. Training data are used for optimization, and validation data are used for model selection. Only the resulting test sets are used for reporting. For example, the 3.2 s eligible test population is 9928 tracklets (3877 fire, 4841 smoke, and 1210 false fire/smoke); applying the overlap rule retains 6896 tracklets (2725 fire, 3422 smoke, and 749 false fire/smoke) for evaluation.

**Figure 5:**
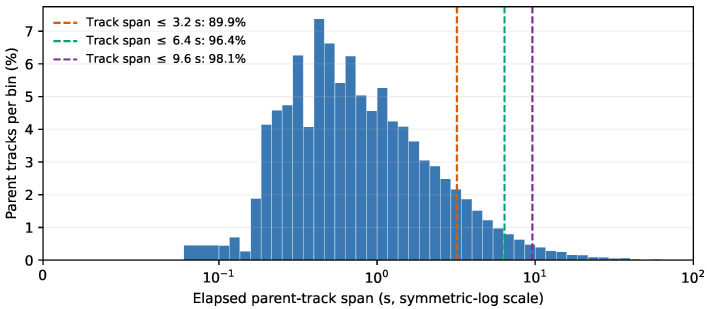
Elapsed-span distribution of the 58,733 parent tracks before duration-specific segmentation. Elapsed span is measured inclusively from the first to the last observation at the recorded source-video frame rate. The symmetric-log horizontal axis is linear near zero and logarithmic thereafter; its 0–100 s range contains 99.96% of the parent tracks. The vertical lines mark the three evaluated temporal contexts.

Overall, the dataset contains 58,733 parent tracks with 2,064,171 annotated track frames from 1347 videos. The parent-track inventory comprises 23,823 fire (40.56%), 28,325 smoke (48.23%), and 6585 false fire/smoke (11.21%) tracks. Track lengths range from 5 to 6879 observed frames, with an average of 35.15±93.20 frames. As shown in Figure 5, track length has a median of 14 frames (interquartile range: 8–33) and a 95th percentile of 120 frames. Measured from the first through the last observation at the source-video frame rate, the median elapsed span is 0.68 s (interquartile range: 0.36–1.48 s), and the 90th and 95th percentiles are 3.24 and 5.17 s. Accordingly, 89.90%, 96.40%, and 98.14% of parent tracks are no longer than the 3.2, 6.4, and 9.6 s contexts, respectively. These bounded contexts retain complete evidence for most tracks while limiting the memory and computation required for the long-tailed remainder. Tracks shorter than five observations are excluded during tracklet construction.

## 5. Experiments

### 5.1. Experimental Setup

This section evaluates STV-FSANet in terms of three-class tracklet verification, false fire/smoke rejection, and early online decision behavior. The pipeline follows the detect–track–verify strategy described in Section 3: a separately trained detector generates candidate regions, association converts them into tracks, and the verifier classifies each sampled track sequence as *fire*, *smoke*, or *false fire/smoke*.

All experiments use the FSAV Tracklet Dataset introduced in Section 4. During dataset preparation, tracks shorter than five frames are removed. For training and evaluation, all sequences are sampled at 10 fps. To study the effect of temporal context length, we use three sequence settings with maximum lengths of 32, 64, and 96 frames, corresponding to 3.2 s, 6.4 s, and 9.6 s of temporal evidence, respectively. We evaluate both matched-context settings, where the dataset subtrack duration matches the model input length, and a fair common-dataset setting, where different sequence lengths are compared using the same 9.6 s source dataset.

STV-FSANet is implemented in PyTorch [47]. Candidate crops are resized to 128×128 and encoded by RepViT-M0.9 [45]; the geometry branch uses the 18-dimensional descriptor in Section 3.3. Single-layer GRUs process both branches before fusion, and recent pooling uses K=12 observations (at most 1.2 s at 10 fps). AdamW uses an initial learning rate and weight decay of $10^{-4}$. Training uses 60 epochs, batch size 4 (2 for 96-frame models), and CUDA automatic mixed precision. Most experiments use seed 42; the matched 3.2 s full model is trained with seeds 42–46 under identical settings. The geometry-only ablation uses 100 epochs and batch size 8. Class-weighted cross-entropy uses inverse class frequencies computed only from the eligible training tracklets and normalized to unit mean; validation and test labels do not affect the weights. For the class-balancing comparison, we additionally evaluate unweighted cross-entropy, focal loss $L_{\mathrm{focal}}=-{\left(1-p_{t}\right)}^{\gamma }\log\left(p_{t}\right)$ with γ=2 [48], and class-balanced inverse-frequency oversampling with replacement. Oversampling retains the original number of training samples per epoch, and neither focal loss nor oversampling is combined with inverse-frequency loss weights. Prefix training is enabled, the appearance backbone is unfrozen after epoch 11, and the checkpoint with the highest validation accuracy is retained.

All training and evaluation experiments were conducted on the same workstation using the environment reported in Table 5. We report both accuracy and macro-F1. For each class *c*,

$$\begin{array}{cc} {\mathrm{Precision}}_{c} & =\frac{TP_{c}}{TP_{c}+FP_{c}},\\ {\mathrm{Recall}}_{c} & =\frac{TP_{c}}{TP_{c}+FN_{c}},\\ F1_{c} & =\frac{2\cdot {\mathrm{Precision}}_{c}\cdot {\mathrm{Recall}}_{c}}{{\mathrm{Precision}}_{c}+{\mathrm{Recall}}_{c}},\\ \mathrm{Macro}\text{-}F1 & =\frac{1}{K}\sum_{c=1}^{K}F1_{c}. \end{array}$$

For the three-class setting in this work,

$$\mathrm{Macro}\text{-}F1=\frac{F1_{\mathrm{fire}}+F1_{\mathrm{smoke}}+F1_{\mathrm{false}\;\mathrm{fire}/\mathrm{smoke}}}{3}.$$

This metric gives equal weight to fire, smoke, and false fire/smoke, reflecting balanced performance on the harder false fire/smoke class rather than being dominated by the more frequent fire and smoke classes.

### 5.2. Main Quantitative Results

**Figure 6:**
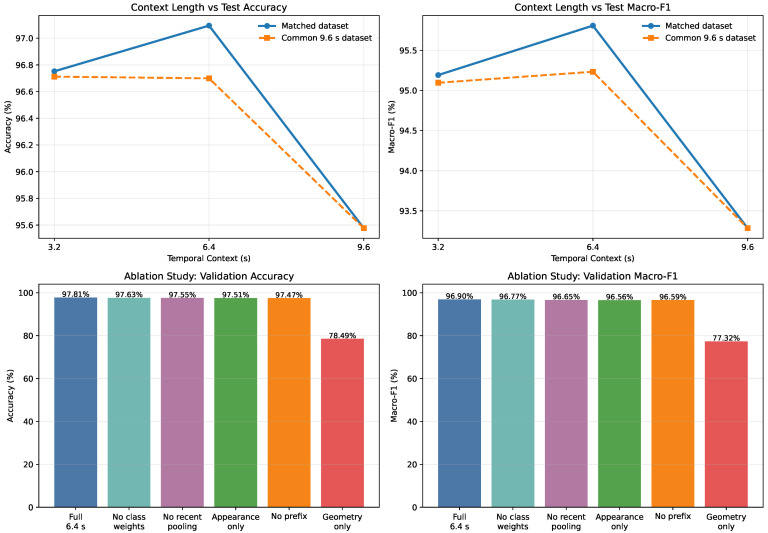
Overview of the experimental results. **Top**: Effect of temporal context length on test accuracy and macro-F1 under matched-context and common-dataset comparisons. **Bottom**: Validation accuracy and validation macro-F1 for 6.4 s ablation variants, reflecting the model-selection criterion used during training.

Table 6 and Figure 6 summarize the main quantitative results. Under the matched-context setting, the 3.2 s model achieves 96.75% test accuracy and 95.19% macro-F1, while the 6.4 s model achieves the best matched-context result, with 97.09% accuracy and 95.81% macro-F1. The 9.6 s setting gives 95.58% accuracy and 93.28% macro-F1. These results indicate that increasing the temporal window does not automatically improve verification performance. In practice, discriminative evidence for fire/smoke verification is often captured within a relatively short temporal horizon, and an excessively long context can introduce redundant or less stable evidence.

To isolate the effect of temporal context length from dataset construction, we further compare different sequence lengths on the same 9.6 s source dataset. In this fair comparison, the 32-frame, 64-frame, and 96-frame models achieve 96.71%, 96.70%, and 95.58% test accuracy, with corresponding macro-F1 scores of 95.10%, 95.23%, and 93.28%, respectively. Thus, both the 3.2 s and 6.4 s settings outperform the 9.6 s setting, with only 0.14 percentage points separating their macro-F1 scores. This result supports the central design hypothesis of the proposed framework: reliable temporal verification does not require very long observation windows but benefits from effective integration of compact temporal evidence.

**Figure 7:**
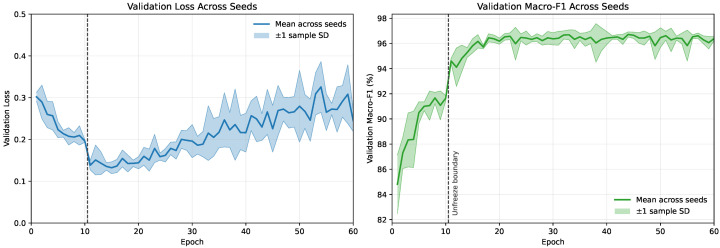
Validation dynamics of the matched 3.2 s model across seeds 42–46. Solid curves show the mean, and shading indicates ±1 sample standard deviation. The dashed line marks backbone unfreezing at epoch 11.

Figure 7 shows the validation dynamics of five matched 3.2 s runs. Validation loss fluctuates after the appearance backbone is unfrozen at epoch 11, so loss alone does not establish overfitting; this behavior can also reflect stochastic optimization with batch size 4. In contrast, validation macro-F1 remains closely clustered. The checkpoints selected by validation accuracy occur at epochs 29–45, yet on the test set the five runs achieve 96.82±0.14% accuracy and 95.34±0.24% macro-F1 (mean ± sample standard deviation). Fire, smoke, and false fire/smoke recall are 97.42±0.23%, 97.76±0.37%, and 90.39±1.50%, respectively. These results show stable performance across the five seeds and selected epochs.

### 5.3. Ablation Study

The ablation results in Table 6 clarify the contribution of each major design choice, while Figure 6 visualizes the validation-side trends used for checkpoint selection. First, the branch ablation shows that, as expected, appearance modeling provides the majority of the discriminative power. The appearance-only model achieves 96.53% test accuracy and 94.86% macro-F1, which remains close to the strongest full-model results. In contrast, the geometry-only model drops sharply to 77.06% accuracy and 74.77% macro-F1. This large gap is consistent with the role of the geometry stream in STV-FSANet: it is designed as a lightweight auxiliary cue source rather than as a standalone recognizer. Its purpose is to complement appearance with motion, scale, and instability cues that can support the decision when visual evidence is ambiguous. The full dual-branch model performs best, indicating that these low-cost geometric cues provide useful support even though they are not the dominant source of discrimination.

Table 7 further isolates the three geometric feature families using geometry-only leave-one-family-out models. Removing static position/shape causes the largest decrease, reducing macro-F1 by 7.41 points and false fire/smoke recall by 14.42 points. Removing short-window fluctuation reduces macro-F1 by 1.10 points and false fire/smoke recall by 3.58 points, showing that recent box instability contributes to false-candidate rejection. First-order motion/change gives a smaller overall contribution: its removal reduces accuracy and macro-F1 by 1.41 and 1.62 points, respectively, although the class-wise effect is not uniform. These results show that all three families provide complementary information. Because FSAV does not provide blur-specific annotations, the experiment characterizes feature-family contributions rather than performance on a distinct blurred-scene subset.

Second, removing recent-evidence pooling reduces validation macro-F1 from 96.90% to 96.65%, while its test-set result remains nearly identical to the full 6.4 s model. This component nevertheless remains important for continuous online inference. A candidate track may persist for a long time while its visual state changes, for example from visible flame to residual smoke after the flame disappears. If all historical evidence is treated uniformly, older fire cues can slow the decision update. Recent-evidence pooling gives greater influence to the latest track behavior, helping the verifier adapt when the current fire/smoke state differs from earlier observations.

The frozen 3.2 s model was also evaluated on the test set with K=4,8,12,16, and 24. Accuracy was 96.65, 96.77, 96.75, 96.74, and 96.74%, respectively; macro-F1 was 94.95, 95.20, 95.19, 95.18, and 95.14%; and false fire/smoke recall was 87.58, 88.52, 88.52, 88.52, and 88.12%. Performance is stable for K=8–24, supporting the use of K=12.

Third, the training-strategy ablations explicitly examine class imbalance and prefix learning. Removing prefix training reduces validation macro-F1 from 96.90% to 96.59%. We further compare four class-balancing strategies using the same common 9.6 s dataset, 64-frame STV-FSANet configuration, seed, training schedule, and validation-based checkpoint rule; only the loss or training-sampling strategy differs. Table 8 reports the complete comparison.

Relative to weighted cross-entropy, unweighted cross-entropy, focal loss, and oversampling increase test macro-F1 by 0.45, 0.45, and 0.46 percentage points, respectively, and improve false fire/smoke recall by 2.98, 1.67, and 3.10 points. Oversampling gives the highest validation macro-F1 (97.33%) and false fire/smoke recall (89.63%), whereas focal loss gives the highest test accuracy (97.12%) and fire recall (98.25%). Thus, no strategy dominates every metric: the alternatives provide modest and complementary gains, while the principal conclusions about the STV-FSANet architecture and temporal context remain unchanged.

The no-prefix ablation is also attractive from a training-efficiency perspective. In our runs, removing prefix sampling reduced the number of training batches per epoch substantially and shortened epoch time from roughly 37–47 min for the prefix-based full models to about 20 min, at the cost of a modest drop in test performance. This suggests that prefix-based training is especially valuable when early online verification quality is prioritized.

### 5.4. Representation Visualization

**Figure 8:**
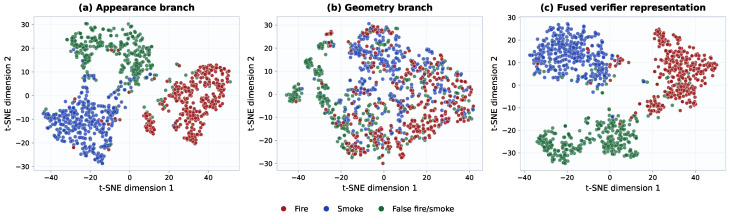
t-SNE visualization of STV-FSANet representations on a balanced subset of 999 test tracklets. Appearance, geometry, and fused verifier representations are projected separately. The fused representation provides the clearest separation of fire, smoke, and false fire/smoke tracklets, consistent with the quantitative ablation results.

To further inspect the learned feature organization, we visualize track-level embeddings from the test split using t-SNE [49]. We use a balanced subset of 999 test tracklets, with 333 samples from each class, and project the appearance, geometry, and fused verifier representations separately. As shown in Figure 8, the fused representation forms the clearest class separation, while the geometry branch alone shows stronger overlap among fire, smoke, and false fire/smoke samples. This supports the ablation finding that appearance provides the dominant discriminative signal and geometry acts as a complementary temporal cue. Because t-SNE [49] is nonlinear, we treat this plot as diagnostic evidence rather than as an additional quantitative metric.

### 5.5. Early Online Verification Analysis

**Figure 9:**
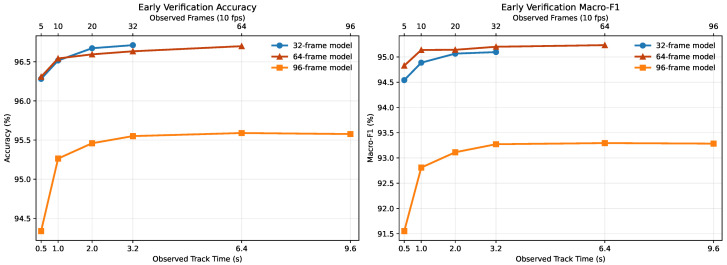
Early online verification behavior on the common 9.6 s test set. The linear bottom axis shows elapsed track time in seconds from the first tracked candidate frame, and the top axis shows the corresponding observed frames at 10 fps. STV-FSANet begins issuing predictions after 5 frames (0.5 s). For both the 32-frame and 64-frame models, accuracy and macro-F1 are within 0.5 percentage points of their final values by 1.0 s; the 96-frame model improves more gradually and remains below the shorter models at full context.

An important objective of the proposed method is to support early and progressively refined online decisions. To evaluate this behavior, we perform prefix-length analysis by truncating each test track to different temporal prefixes and re-evaluating the verifier. Figure 9 summarizes the results for the full models trained on the common 9.6 s dataset. To make the online-decision behavior easier to interpret, the horizontal axis shows elapsed track time at 10 fps, while the secondary axis reports the corresponding number of observed frames. Thus, the figure can be read directly as a response-time plot from the beginning of the tracked candidate sequence.

The 32-frame model reaches 96.28% accuracy and 94.54% macro-F1 at 0.5 s, increasing to 96.52% and 94.89% at 1.0 s; its final values are 96.71% and 95.10%. The 64-frame model similarly reaches 96.31%/94.83% at 0.5 s and 96.54%/95.14% at 1.0 s, compared with final 96.70%/95.23%. Thus, using a prespecified 0.5-percentage-point tolerance for both metrics, both models reach near-final performance by 1.0 s. In contrast, the 96-frame model improves from 94.34%/91.55% at 0.5 s to 95.58%/93.28% at 9.6 s and remains below the shorter-context models.

### 5.6. Class-Wise Error Analysis

**Figure 10:**
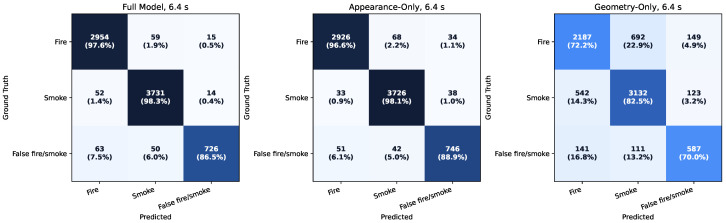
Row-normalized confusion matrices for the full, appearance-only, and geometry-only 6.4 s verification models on the same common 9.6 s test set. The full dual-branch model gives the highest overall accuracy and macro-F1, while the appearance-only model is close and gives slightly higher false fire/smoke recall. The geometry-only variant shows substantially greater confusion across all three classes.

Figure 10 provides a class-wise view of the verification behavior. All three panels use the same 7664-tracklet test set and 6.4 s input. The full model correctly classifies 2954/3028 fire tracks, 3731/3797 smoke tracks, and 726/839 false fire/smoke tracks, corresponding to recalls of 97.6%, 98.3%, and 86.5%, respectively. The appearance-only model remains close and has slightly higher false fire/smoke recall, correctly classifying 746/839 tracks (88.9%), although the full model makes fewer errors overall and achieves the higher accuracy and macro-F1. Thus, geometry does not improve every class recall, but it improves the overall balanced decision. In contrast, the geometry-only model exhibits much stronger confusion across all three classes, and its false fire/smoke recall drops to 70.0%. Geometric evolution alone is therefore insufficient for false-alarm suppression and instead serves as an auxiliary cue that complements appearance-based verification.

### 5.7. Qualitative Results

**Figure 11:**
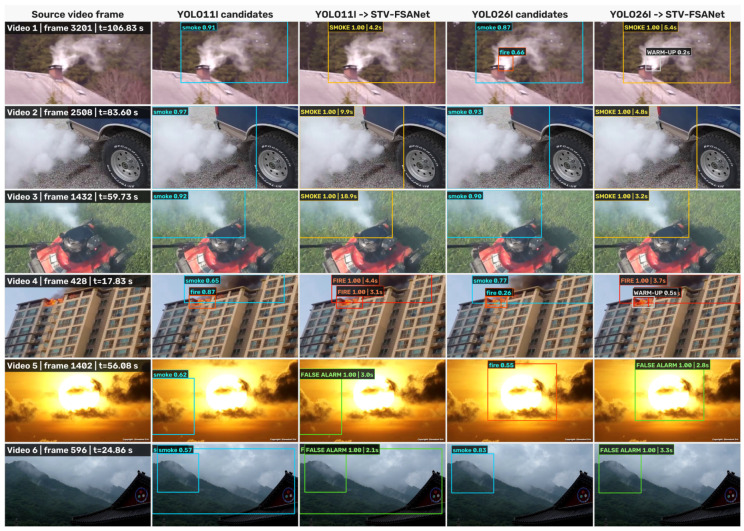
Paired qualitative examples using two detectors. Each row shows one video at a matched timestamp: the source frame, YOLO11l candidates and their STV-FSANet verification, and YOLO26l candidates and their STV-FSANet verification. Both paths use the same association settings and frozen verifier; the displayed decisions use the accumulated track history rather than only the shown frame.

To examine detector replacement at the candidate-track interface, Figure 11 compares YOLO11l–STV-FSANet and YOLO26l–STV-FSANet on the same videos and timestamps, using the same association settings and frozen verifier. The detectors can produce different candidate regions, as shown in the figure, while STV-FSANet independently verifies the temporally associated crop sequence. Accordingly, end-to-end pipeline performance depends on the upstream detector localizing the relevant candidate regions accurately enough to form usable tracks; missed or poorly localized regions cannot be recovered by the verifier.

Although the detector reports fire/smoke labels and confidence scores, these values are displayed only to make frame-level behavior visible. The verifier uses the expanded candidate boxes for crop extraction and geometric cues, then produces the final fire, smoke, or false fire/smoke decision from the associated track. Thus, it can be paired with candidate detectors using any class taxonomy as long as their boxes can be temporally associated. Detector labels could be used as an optional association cue, but the qualitative examples use class-agnostic association.

The examples cover true fire scenes, true smoke scenes, and visually confusing false-alarm scenes. New tracks remain in a warm-up state until the minimum temporal evidence is available, avoiding premature alarms. Once sufficient evidence has accumulated, persistent fire and smoke candidates are validated, while many ambiguous or transient detector responses are classified as false fire/smoke class. This shows that STV-FSANet assigns alarms from accumulated appearance and geometric evidence rather than forwarding instantaneous detector outputs.

### 5.8. Contextual Comparison with Representative Prior Methods

Table 9 summarizes representative frame-level detectors, clip-level classifiers, spatio-temporal models, and post-detection decision methods using the results reported by their source papers. Because their tasks, datasets, splits, label spaces, and metrics differ, the numerical values provide literature context rather than a controlled ranking. DTA/DTAIF [7,8] are conceptually related through post-detection temporal evidence accumulation, whereas FOCUS [17], LwST-FSD [14], and other entries address different detection or recognition settings. STV-FSANet is distinguished here by its three-class candidate-track verification formulation and explicit false fire/smoke rejection.

Table 10 uses the same test tracklets, 10 fps sampling, and context limits for both methods. Majority-vote ties are resolved by summed detector confidence and then deterministically by class name. STV-FSANet improves shared-output-space accuracy by 7.03–8.24 percentage points and macro-F1 by 8.18–9.09 points. Unlike detector voting, it also supports explicit false fire/smoke rejection.

Overall, the experiments support the design of STV-FSANet. Short-to-moderate temporal contexts outperform very long observation windows; the appearance branch provides most of the discriminative power; and the geometry branch acts as a lightweight stabilizing cue. Prefix-based evaluation further confirms that strong decisions can be made early in the track, which is essential for continuous surveillance.

### 5.9. End-to-End Online Efficiency Analysis

We evaluate the end-to-end online inference efficiency of the complete alarm-validation pipeline, including YOLO11l frame-level candidate localization, online temporal association, and STV-FSANet track verification. In this benchmark, YOLO11l operates at an input size of 640×640, detections are mapped back to the original frame resolution for tracking and crop extraction, and the STV-FSANet verifier processes resized 128×128 candidate crops. To match the training setup, the verifier samples track observations at 10 fps and begins issuing online decisions when the fifth sampled observation becomes available. At each video frame, all active tracks satisfying this decision condition are processed together as a verifier batch, while the final alarm state is still assigned separately for each track. The reported runs use class-aware association with $\tau_{\mathrm{IoU}}=0.30$ and $M_{\mathrm{miss}}=5$; hence, a track is retained through five consecutive misses and retired on the sixth. Runtime is measured on six representative source videos, one from each constituent source dataset. Latency and throughput are first summarized within each video and then aggregated across the six videos. Consequently, the reported mean throughput is $\frac{1}{6}\sum_{i}1000/L_{i}$, not the reciprocal $1000/(\frac{1}{6}\sum_{i}L_{i})$ of the displayed mean latency. For the Windows TensorRT run, these quantities are 77.88 and 72.13 frames/s, respectively. The complete runtime results are reported in Table 11.

Table 11 compares PyTorch [47] and TensorRT FP16 [51] inference on three evaluation platforms. On the Windows 10 Workstation evaluated through WSL2 Ubuntu 20.04 with an RTX 4080 GPU, TensorRT reduces total latency from 26.35 ms/frame to 13.86 ms/frame and increases throughput from 38.92 to 77.88 frames/s. On the Ubuntu Compact PC with a Core Ultra 7 155H CPU and RTX 4060 Laptop GPU, TensorRT reduces total latency from 16.93 ms/frame to 9.39 ms/frame and increases throughput from 59.74 to 109.55 frames/s. On the NVIDIA Jetson AGX Orin DevKit, TensorRT reduces total latency from 64.43 ms/frame to 25.91 ms/frame and increases throughput from 15.78 to 39.51 frames/s. The association stage remains below 0.1 ms/frame in all settings, indicating that most computation is concentrated in the detector and temporal verifier. The benchmark also includes a multi-track online workload, with approximately 3.1 active tracks per frame on average and up to 40 active tracks in the most crowded video. These results show that the proposed detect–track–verify pipeline is practical for real-time surveillance, including edge deployment when TensorRT acceleration is available.

Table 12 distinguishes stored artifacts from operational memory. Runtime memory was measured on the Jetson AGX Orin during the six-video workload; the reported process and unified system memory measurements are complementary and are therefore not additive.

## 6. Discussion

The experimental results support the main premise of this paper: reliable fire and smoke alarms benefit from temporal verification beyond frame-level detection alone. Across both matched-context and common-dataset settings, STV-FSANet performs best with compact temporal contexts. This indicates that useful alarm-verification evidence is often accumulated early and that longer temporal spans can add redundant or stale observations without improving the final decision.

The ablation, confusion-matrix, and qualitative results further show where the improvement comes from. The appearance stream provides the dominant signal, while the geometry stream acts as a lightweight complementary cue. Recent-evidence pooling also matches the online task: even when its numerical gain is modest, it helps the verifier emphasize the current state of a long-running track. Class-wise analysis shows strong false fire/smoke rejection, and the qualitative examples demonstrate that transient or ambiguous detector responses can be rejected after temporal evidence is accumulated. The controlled class-balancing comparison shows that unweighted cross-entropy, focal loss, and class-balanced oversampling each improve test macro-F1 over inverse-frequency weighted cross-entropy by approximately 0.45–0.46 percentage points. Oversampling provides the highest false fire/smoke recall, while focal loss provides the highest fire recall and overall test accuracy; consequently, no single balancing strategy is uniformly superior.

Prefix-based evaluation is particularly important for the alarm-validation setting. Both the 32-frame and 64-frame models are within 0.5 percentage points of their final accuracy and macro-F1 by 1.0 s and begin issuing predictions after only 0.5 s. This early-response behavior is a key advantage for online surveillance systems, where the timeliness of an alarm can be as important as the final classification score. DTA/DTAIF report observation times under different evaluation protocols, so these values are not directly comparable.

The end-to-end efficiency results support the practicality of the staged design. Since association contributes negligible overhead, the runtime is governed mainly by the frame-level detector and the STV-FSANet verifier. TensorRT FP16 acceleration substantially reduces both components, improving throughput from 59.74 to 109.55 frames/s on the Ubuntu Compact PC with an RTX 4060 Laptop GPU and from 15.78 to 39.51 frames/s on the NVIDIA Jetson AGX Orin DevKit. These results suggest that the verifier is feasible as part of a complete online alarm-validation pipeline on compact PCs and embedded edge devices.

The main practical limitation is the dependence on upstream localization and association: missed boxes cannot be recovered, while poorly localized boxes or fragmented tracks degrade the verifier’s evidence. When association breaks, a later candidate receives a new track ID and is verified as an independent fragment; its recurrent states and recent-evidence buffers are reinitialized because the verifier does not perform fragment stitching or re-identification. A decision becomes available at the fifth sampled observation and is updated after each later observation. Splitting can therefore delay an alarm, shorten the usable appearance history, interrupt the geometric trajectory, or leave very short fragments unverified. Fixed-tracklet metrics characterize correctly localized and associated candidates, whereas end-to-end coverage also depends on detector recall and association continuity. Stronger association, fragment stitching, or state handover between track fragments could mitigate these effects in deployment. Their systematic comparison, broader generalization studies, and mixture-aware fire–smoke annotations remain future work.

## 7. Conclusions

This paper presented STV-FSANet, a spatio-temporal verification framework for fire and smoke alarm validation in real-world video surveillance. Instead of relying on instantaneous detector outputs, the proposed detect–track–verify pipeline uses a frame-level detector to localize candidate regions, associates them into tracks, and classifies each observed track sequence as fire, smoke, or false fire/smoke. This modular design separates alarm validation from the detector backbone, allowing future improvements in candidate localization to be incorporated without redesigning the temporal decision module. To support this formulation, we also constructed the FSAV Tracklet Dataset from five public video sources and one source obtained directly from the original authors, comprising 1347 videos, 58,733 parent tracks, and 2.06 million annotated track frames.

The experimental results show that STV-FSANet provides accurate, early, and deployable alarm validation. The best matched-context model achieves 97.09% accuracy and 95.81% macro-F1, while the common-dataset comparison shows that compact 32-frame and 64-frame contexts are more effective than a longer 96-frame context. Prefix-based evaluation further shows that the shorter models come within 0.5 percentage points of their final metrics by 1.0 s, which is essential for online surveillance. The class-wise and qualitative results also confirm effective rejection of false fire/smoke tracks in visually confusing scenes.

Finally, the end-to-end runtime analysis confirms that the verification system can operate as part of a complete online alarm-validation pipeline. With TensorRT FP16 acceleration, the deployed YOLO11l–STV-FSANet pipeline reaches 109.55 frames/s on an Ubuntu Compact PC with an RTX 4060 Laptop GPU and 39.51 frames/s on an NVIDIA Jetson AGX Orin DevKit. Overall, robust fire/smoke alarm generation does not require very long observation periods; compact track-level temporal verification can support rapid response, reliable false-alarm suppression, and practical deployment on real surveillance hardware.

## Figures and Tables

**Table 1:** Lightweight geometric temporal cues computed from each tracked candidate box. The cues are grouped by role to make the auxiliary geometry stream compact and interpretable.

Feature Family	Feature Group	Feature Definition	Purpose
Static position/shape	Normalized center	$\begin{array}{c} cx_{n}^{\left(t\right)}=\frac{x_{1}^{\left(t\right)}+x_{2}^{\left(t\right)}}{2W},\;cy_{n}^{\left(t\right)}=\frac{y_{1}^{\left(t\right)}+y_{2}^{\left(t\right)}}{2H} \end{array}$	Candidate location and drift reference.
	Normalized size	$\begin{array}{c} w_{n}^{\left(t\right)}=\frac{x_{2}^{\left(t\right)}-x_{1}^{\left(t\right)}}{W},\;h_{n}^{\left(t\right)}=\frac{y_{2}^{\left(t\right)}-y_{1}^{\left(t\right)}}{H} \end{array}$	Box scale independent of image resolution.
	Area and aspect	$\begin{array}{c} area_{n}^{\left(t\right)}=\frac{\left(x_{2}^{\left(t\right)}-x_{1}^{\left(t\right)}\right)\left(y_{2}^{\left(t\right)}-y_{1}^{\left(t\right)}\right)}{WH},\;aspect^{\left(t\right)}=\frac{x_{2}^{\left(t\right)}-x_{1}^{\left(t\right)}}{y_{2}^{\left(t\right)}-y_{1}^{\left(t\right)}+\epsilon } \end{array}$	Coarse region extent and shape.
First-order motion/change	Center displacement	$dcx^{\left(t\right)}=cx_{n}^{\left(t\right)}-cx_{n}^{\left(t-1\right)},dcy^{\left(t\right)}=cy_{n}^{\left(t\right)}-cy_{n}^{\left(t-1\right)}$	Frame-to-frame candidate motion.
	Scale and shape change	$\begin{array}{c} dw^{\left(t\right)}=w_{n}^{\left(t\right)}-w_{n}^{\left(t-1\right)},\;dh^{\left(t\right)}=h_{n}^{\left(t\right)}-h_{n}^{\left(t-1\right)},\\ darea^{\left(t\right)}=area_{n}^{\left(t\right)}-area_{n}^{\left(t-1\right)},\;daspect^{\left(t\right)}=aspect^{\left(t\right)}-aspect^{\left(t-1\right)} \end{array}$	Growth, shrinkage, and deformation over time.
	Motion trend	$\begin{array}{c} motion\_mag^{\left(t\right)}=\sqrt{{\left(dcx^{\left(t\right)}\right)}^{2}+{\left(dcy^{\left(t\right)}\right)}^{2}},\;upward\_motion^{\left(t\right)}=-dcy^{\left(t\right)} \end{array}$	Overall displacement and upward drift cue.
Short-window fluctuation	Recent fluctuation	$\begin{array}{c} std\_area_{k}^{\left(t\right)}=\mathrm{Std}\left(area_{n}^{\left(t-k+1:t\right)}\right),\;std\_cx_{k}^{\left(t\right)}=\mathrm{Std}\left(cx_{n}^{\left(t-k+1:t\right)}\right),\\ std\_cy_{k}^{\left(t\right)}=\mathrm{Std}\left(cy_{n}^{\left(t-k+1:t\right)}\right),\;std\_aspect_{k}^{\left(t\right)}=\mathrm{Std}\left(aspect^{\left(t-k+1:t\right)}\right) \end{array}$	Short-term instability over the recent *k*-frame window, where $q^{\left(t-k+1:t\right)}$ denotes recent values.

**Table 2:** Five publicly accessible fire/smoke video sources and one source obtained directly from its authors used to construct the FSAV Tracklet Dataset. The table summarizes the source annotation status, dataset scale, primary role, and acquisition source of each dataset used during curation.

Dataset Source	Original Annotation	Source Videos and Content	Use in Curation	Access/ Origin
FIRESENSE (2017) [41]	No	**49 videos** (Flame: 11 positive + 16 negative; Smoke: 13 positive + 9 negative)	Flame and smoke benchmark	https://zenodo.org/records/836749#.XF87T7nVLIU (accessed on 17 March 2026)
Furg Fire Dataset (2017) [32]	Yes	**23 videos** (annotated flame videos with XML bounding boxes)	Fire/flame detection with XML boxes	https://github.com/steffensbola/furg-fire-dataset (accessed on 19 March 2026)
KMU Fire & Smoke Dataset (2012) [29]	No	**38 videos** (22 indoor/outdoor short-distance flame; 2 indoor/outdoor short-distance smoke; 10 smoke/flame-like moving objects; 4 wildfire smoke)	Flame, smoke, wildfire smoke, and hard negatives	https://cvpr.kmu.ac.kr/Dataset/Dataset.htm (accessed on 19 March 2026)
Bilkent VisiFire Sample Clips [42]	No	**32 videos** (13 fire; 19 smoke sample clips)	Fire and smoke sample clips	https://kilyos.ee.bilkent.edu.tr/~signal/VisiFire/ (accessed on 19 March 2026)
SmokeNet-ProjectX (2020) [25]	Yes	**678 videos** (raw labeled videos; also provides extracted frames and derived grid labels)	Wildfire smoke detection	https://archive.org/details/smokenet-projectx (accessed on 6 April 2026)
Kim–Lee Fire Video Dataset (2019) [7,8]	Yes (Partially)	**1484 received videos**; 617 selected fire, smoke, and non-fire clips	Deep learning-based video fire detection with temporal modeling	Author-provided collection used here; obtained directly from the original authors

**Table 3:** Parent-track inventory used to construct the FSAV Tracklet Dataset. For each source dataset, videos were curated by removing duplicates, excluding unsuitable samples, and keeping only videos that produced valid final tracks. The table reports the number of selected videos, class-wise track counts, total tracks, average track length (mean±std), and track length range after final validation.

Dataset	Selected Videos	Fire Tracks	Smoke Tracks	False Fire/Smoke Tracks	Total Tracks	Average Track Length (Frames)	Track Length Range (Frames)
FIRESENSE (2017) [41]	34	69	237	618	924	22.16±41.15	5–683
Furg Fire Dataset (2017) [32]	22	939	106	95	1140	30.08±49.89	5–780
KMU Fire & Smoke Database (2012) [29]	36	1331	441	343	2115	47.45±93.65	5–1897
Bilkent VisiFire Sample Clips [42]	29	509	324	2	835	34.61±62.25	5–711
SmokeNet-ProjectX (2020) [25]	609	6995	19,266	835	27,096	32.94±97.09	5–6191
Kim-Lee Fire Video Dataset (2019) [7,8]	617	13,980	7951	4692	26,623	37.09±92.50	5–6879
**Overall**	**1347**	**23,823**	**28,325**	**6585**	**58,733**	35.15±93.20	**5–6879**

**Table 4:** Duration-specific FSAV tracklet populations by split. Eligible counts are obtained after 10 fps sampling and application of the five-observation minimum. Test counts after filtering exclude tracklets that share any sampled candidate-region observation or parent-track identity with training or validation data.

Context	Nominal Subtracks			Eligible at 10 fps			Test After Filtering
	Train	Val.	Test	Train	Val.	Test	
3.2 s (32 frames)	44,779	10,333	13,780	32,185	7484	9928	6896
6.4 s (64 frames)	40,540	9354	12,478	27,969	6505	8603	7502
9.6 s (96 frames)	39,374	9085	12,118	26,813	6197	8272	7664

**Table 5:** Training platform and reproducibility environment.

Item	Configuration
Workstation	Intel Core i9-11900K (8 cores/16 threads), 64 GB system memory
GPU	NVIDIA GeForce RTX 4080, 16 GB GPU memory
Operating system	Windows 11 Pro, 64-bit
Core software	Python 3.13.14; PyTorch 2.12.1; CUDA 12.6; cuDNN 9.10.2
Model/evaluation libraries	torchvision 0.27.1; timm 1.0.28; scikit-learn 1.9.0

**Table 6:** Summary of experimental results. All values are reported in %. The table is organized into two blocks: a context-length comparison for the full STV-FSANet model and a 6.4 s ablation study. “Evaluation setting” combines the dataset build and protocol, while “temporal input” reports the maximum sequence length used by the verifier. Test values use the test populations defined in Section 4.3.

Model Variant	Evaluation Setting	Temporal Input	Val. Acc.	Val. Macro-F1	Test Acc.	Test Macro-F1
**Context-Length Comparison (Full STV-FSANet)**						
Full STV-FSANet	Matched 3.2 s dataset	3.2 s (32 frames)	**97.74**	**96.95**	96.75	95.19
Full STV-FSANet	Matched 6.4 s dataset	6.4 s (64 frames)	97.63	96.85	**97.09**	**95.81**
Full STV-FSANet	Matched 9.6 s dataset	9.6 s (96 frames)	96.26	94.87	95.58	93.28
Full STV-FSANet	Common 9.6 s dataset	3.2 s (32 frames)	97.60	96.66	96.71	95.10
Full STV-FSANet	Common 9.6 s dataset	6.4 s (64 frames)	97.81	96.90	96.70	95.23
Full STV-FSANet	Common 9.6 s dataset	9.6 s (96 frames)	96.26	94.87	95.58	93.28
**Ablation Study (Common 9.6 s Dataset, 6.4 s Input)**						
Full STV-FSANet (reference)	Common 9.6 s dataset	6.4 s (64 frames)	97.81	96.90	96.70	95.23
Appearance-only	Common 9.6 s dataset	6.4 s (64 frames)	97.51	96.56	96.53	94.86
Geometry-only	Common 9.6 s dataset	6.4 s (64 frames)	78.49	77.32	77.06	74.77
No prefix training	Common 9.6 s dataset	6.4 s (64 frames)	97.47	96.59	96.40	94.59
No recent pooling	Common 9.6 s dataset	6.4 s (64 frames)	97.55	96.65	96.70	95.21
No class weighting	Common 9.6 s dataset	6.4 s (64 frames)	97.63	96.77	97.05	95.69

*Note*: “**Matched**” means that the dataset subtrack duration used during construction is the same as the verifier input duration (e.g., 3.2 s tracklets evaluated with 3.2 s input). “**Common 9.6 s dataset**” means that all experiments use tracklets built from the same 9.6 s source dataset, while the verifier input length varies for a fair comparison or ablation.

**Table 7:** Geometry feature-family ablation on the common 9.6 s test set. Each leave-one-family-out model masks the corresponding dimensions of the feature families defined in Table 1 during training and evaluation. Check marks (✓) identify the retained feature families; all performance values are reported in %.

Experiment	Retained Geometry Feature Families			Test Performance				
	Static Position/Shape	First-Order Motion/Change	Short-Window Fluctuation	Accuracy	Macro-F1	Fire Recall	Smoke Recall	False Fire/ Smoke Recall
Full 18-D geometry features	✓	✓	✓	77.06	74.77	72.23	82.49	69.96
Without static position/shape	–	✓	✓	71.40	67.36	72.36	74.14	55.54
Without first-order motion/change	✓	–	✓	75.65	73.15	69.55	81.51	71.16
Without short-window fluctuation	✓	✓	–	75.67	73.68	73.15	79.72	66.39

**Table 8:** Full-model comparison of class-balancing strategies on the common 9.6 s dataset with 6.4 s (64-frame) input. Validation and test values are reported in %. Bold values indicate the best result at the displayed precision.

Balancing Strategy	Val. Acc.	Val. Macro-F1	Test Acc.	Test Macro-F1	Fire Recall	Smoke Recall	False Fire/ Smoke Recall
Inverse-frequency weighted cross-entropy	97.82	96.93	96.70	95.23	97.56	98.26	86.53
Unweighted cross-entropy	97.61	96.74	97.05	**95.69**	97.46	98.39	89.51
Focal loss (γ=2)	97.93	97.17	**97.12**	95.68	**98.25**	98.18	88.20
Class-balanced oversampling	**97.98**	**97.33**	97.03	**95.69**	97.29	**98.45**	**89.63**

**Table 9:** Contextual literature summary of representative prior methods. Values are reproduced in the form reported by the source papers and are not directly comparable because the tasks, datasets, splits, label spaces, and metrics differ.

Method	Formulation	Dataset/Setting	Main Result(s)
DTA (2019) [7]	Binary video fire decision with Faster R-CNN, LSTM, and majority voting	Large custom fire videos + public 31-video fire benchmark	95.0% short-term clip accuracy; 99.64% at 1.5 min; 100% at 2.5–3 min
DTAIF (2021) [8]	Binary video fire decision with Bayesian fusion over DTA outputs	Public fire benchmark with 30 s–3 min decision intervals	97.6%, 98.5%, and 99.3% at 30, 60, and 90 s; 100% at 2 min
EfficientDet Smoke Detector (2023) [10]	Frame-level wildfire smoke localization	14,125 smoke and 21,203 non-smoke images	True detection rate 80.4%; false-positive rate 1.13%
3DVSD (2022) [22]	End-to-end 3D video smoke detection/localization	Sequential smoke video benchmark	Accuracy 99.54%; false alarm 1.11%; missed detection 0.14%
FireMatch (2023) [40]	Semi-supervised clip-level video fire classification	Two real-world fire video datasets	Accuracy 76.92% and 91.80% on two separate datasets
STENet (2024) [23]	Spatio-temporal smoke recognition with short- and long-term modeling	RISE industrial smoke and FSmoke forest-smoke benchmarks	F-score 0.893 on RISE; 6.9M params; 3.86 GFLOPs
YOLOFM (2024) [13]	Frame-level fire/smoke object detection based on YOLOv5n	FM-VOC dataset (18,644 images)	+3.1 Prec., +3.9 Rec., +3.0 F1, +2.2 mAP@0.5, +7.9 mAP@0.5:0.95 vs. YOLOv5n
FOCUS (2025) [17]	Scenario-adaptive video fire detection	ONFIRE 2023 + FIRE-TASTIC	96.68% F, 98.08% P, 95.53% R, 0.6711 FDS on ONFIRE; 98.08% F on FIRE-TASTIC
LwST-FSD (2025) [14]	Lightweight spatio-temporal smoke detection	FIgLib, SKLFS-WildFire, SKLFS-WildFire2025	87.42% acc. and 87.36% F1 on FIgLib; 0.330 AP@0.5, 0.832/0.822 image/video AUC on SKLFS-WildFire2025
Hybrid ViT–3D-CNN (2026) [50]	ViT+3D-CNN fire/smoke detection	NASA + Fire Videos	99.2% acc. (NASA), 98.3% acc. (Fire Videos), 32/18 frames/s (GPU/Jetson NX)
**STV-FSANet (ours)**	**Track-level three-class alarm validation after detect–track association**	**1347 videos, 58,733 parent tracks, 2.06M annotated track frames**	**97.09% test accuracy, 95.81% macro-F1; near-final prefix metrics by 1.0 s; 39.51 frames/s end-to-end on NVIDIA Jetson AGX Orin DevKit**

**Table 10:** Controlled same-data comparison with YOLO11l temporal majority voting (YOLO11l-MV). Metrics are computed on the same ground-truth fire/smoke tracklets because YOLO11l has no false fire/smoke output. STV-FSANet predictions of false on these positive tracks are counted as errors. False-class recall is reported separately and is not applicable (N/A) to majority voting.

Context	Fire/Smoke Tracks	Fire/Smoke Accuracy (%)		Fire/Smoke Macro-F1 (%)		False Fire/Smoke Recall (%)	
		YOLO11l-MV	STV-FSANet	YOLO11l-MV	STV-FSANet	YOLO11l-MV	STV-FSANet
3.2 s	6147	89.80	97.76	89.34	98.13	N/A	88.52
6.4 s	6663	89.72	97.96	89.21	98.30	N/A	90.23
9.6 s	6825	89.79	96.82	89.33	97.52	N/A	85.46

**Table 11:** End-to-end online efficiency of the complete YOLO11l detection–association–STV-FSANet verification pipeline. Values are reported as mean ± standard deviation over six representative source videos. Latency is reported in milliseconds per frame (ms/frame), and throughput is reported in frames per second (frames/s). For video *i*, throughput is computed as $1000/L_{i}$, where $L_{i}$ is that video’s mean total pipeline latency; the table then reports the mean and standard deviation of the six per-video throughput values. The active-track column reports the mean number of tracks per frame and the peak number of active tracks observed across the benchmark videos; eligible active-track sequences are processed as a verifier batch at each frame.

Platform	Runtime Environment.	Backend	YOLO11l ms/Frame	Assoc. ms/Frame	STV-FSANet ms/Frame	Total ms/Frame	Throughput Frames/s	Tracks Mean/Peak
Windows 10 WS	WSL2 Ubuntu 20.04 RTX 4080	PyTorch	14.68 ± 1.01	0.039 ± 0.011	11.64 ± 4.79	26.35 ± 4.77	38.92 ± 6.50	3.14 ± 1.39/40
Windows 10 WS	WSL2 Ubuntu 20.04 RTX 4080	TensorRT FP16	7.69 ± 1.08	0.041 ± 0.012	6.13 ± 3.39	13.86 ± 4.36	77.88 ± 22.25	3.15 ± 1.41/38
Ubuntu Compact PC	Ubuntu 24.04 LTS RTX 4060 Laptop	PyTorch	10.51 ± 0.96	0.024 ± 0.008	6.39 ± 2.62	16.93 ± 2.04	59.74 ± 6.84	3.14 ± 1.39/40
Ubuntu Compact PC	Ubuntu 24.04 LTS RTX 4060 Laptop	TensorRT FP16	5.69 ± 0.30	0.023 ± 0.008	3.68 ± 1.88	9.39 ± 1.78	109.55 ± 19.54	3.16 ± 1.42/39
Jetson AGX Orin DevKit	Ubuntu 20.04 JetPack 5.1.2	PyTorch	39.18 ± 1.96	0.086 ± 0.024	25.16 ± 10.40	64.43 ± 9.42	15.78 ± 2.11	3.14 ± 1.38/40
Jetson AGX Orin DevKit	Ubuntu 20.04 JetPack 5.1.2	TensorRT FP16	15.85 ± 1.56	0.085 ± 0.026	9.98 ± 5.24	25.91 ± 4.45	39.51 ± 6.49	3.16 ± 1.41/40

CPU details: Windows 10 Workstation: Intel(R) Core(TM) i9-11900K @ 3.50 GHz; Ubuntu Compact PC: Intel(R) Core(TM) Ultra 7 155H × 22; NVIDIA Jetson AGX Orin DevKit: 12-core ARMv8 CPU.

**Table 12:** Deployed-model complexity, storage, and embedded-device memory. YOLO11l complexity is measured per 640×640 frame, whereas STV-FSANet is measured per online update of one 128×128 candidate-region observation and its recurrent state. PyTorch weight sizes are serialized FP32 model states. TensorRT sizes are serialized FP16 inference engines built separately on each indicated platform. Operational memory was measured during a repeat of the six-video workload on the Jetson AGX Orin DevKit in MAXN mode.

*Model complexity and stored artifacts*					
**Component**	**GFLOPs**	**Parameters** **(million)**	**PyTorch Weights** **(MiB)**	**TensorRT FP16 (MiB)**	
				**RTX 4080**	**Jetson Orin**
YOLO11l	87.28	25.31	97.09	51.70	51.59
STV-FSANet	0.55	5.34	20.82	11.93	11.85
*Jetson AGX Orin DevKit operational memory (MiB)*					
**Backend**	**Precision**	**Peak process** **memory**	**Baseline system** **memory**	**Peak system** **memory**	**Increase**
PyTorch	FP32	3660.39	13,737	17,343	3606
TensorRT	FP16	3949.65	13,689	17,228	3539

## Data Availability

Five third-party source collections used in this study are publicly available through the citations and URLs provided in Table 2. The Kim–Lee Fire Video Dataset was obtained directly from its original authors. Restrictions apply to this collection; the present authors do not have permission to redistribute it, and access must be requested from the original provider. The derived FSAV tracklet annotations, data splits, and other data supporting the reported results are available from the corresponding author upon reasonable request, subject to the terms and redistribution restrictions of the source datasets.
